# Level of Postnatal Checkup in Ethiopia – Implications for Child Health Services

**DOI:** 10.3389/fped.2022.895339

**Published:** 2022-06-14

**Authors:** Binyam Minuye Birhane, Wubet Alebachew Bayih, Demewoz Kefale Mekonen, Ermias Sisay Chanie, Solomon Demis, Habtamu Shimelis, Worku Necho Asferie, Eskeziaw Abebe, Dagne Addisu, Gedefaye Nibret, Aklilu Endalamaw, Tigabu Munye, Desalegn Abebaw Jember, Samuel Nebiyu, Yenework Mulu Tiruneh, Demeke Mesfin Belay

**Affiliations:** ^1^College of Health Sciences, Debre Tabor University, Debre Tabor, Ethiopia; ^2^College of Health Sciences, Woldia University, Woldia, Ethiopia; ^3^School of Public Health, The University of Queensland, Brisbane, QLD, Australia; ^4^School of Health Sciences, College of Medicine and Health Sciences, Bahir Dar University, Bahir Dar, Ethiopia; ^5^St. Paul’s Hospital, Addis Ababa, Ethiopia; ^6^College of Medicine and Health Sciences, Wollo University, Dessie, Ethiopia; ^7^College of Medicine and Health Sciences, University of Gondar, Gondar, Ethiopia

**Keywords:** EDHS, Ethiopia, neonatal checkup, postnatal visit, neonate

## Abstract

**Background:**

High neonatal mortality rates continue to be a major public health issue in Ethiopia. Despite different maternal and neonatal care interventions, neonatal mortality in Ethiopia is at a steady state. This could be due to the low utilization of neonatal checkups. Thus, nationally assessing the level and predictors of postnatal checkups could provide important information for further improving neonatal healthcare services.

**Materials and Methods:**

A secondary data analysis of the 2016 Ethiopia Demographic and Health Survey (EDHS) was performed on 7,586 women who had live births in the 2 years before the survey. All variables with a *p*-value of ≤0.25 in the bivariable analysis were entered into the final model for multivariable analysis, and the level of statistical significance was declared at a *P*-value of <0.05.

**Results:**

According to the national survey, only 8.3% [95% CI: 8.19, 8.41] of neonates received postnatal checkups. About two-thirds of women, 62.8% had antenatal care visits, 67.9%, gave birth at home, and 95.7% were unaware of neonatal danger signs. Distance from health care institutions [AOR = 1.42; 95% CI: 1.06, 1.89], giving birth in a healthcare facility [AOR = 1.55; 95% CI: 1.12, 2.15], antenatal care visit [AOR = 3.0; 95% CI: 1.99, 4.53], and neonatal danger signs awareness [AOR = 3.06; 95% CI: 2.09, 4.5] were all associated with postnatal care visits.

**Conclusion:**

The number of neonates who had a postnatal checkup was low. Increasing antenatal care visit utilization, improving institutional delivery, raising awareness about neonatal danger signs, increasing access to health care facilities, and implementing home-based neonatal care visits by healthcare providers could all help to improve postnatal checkups.

## Introduction

The postnatal period is defined as the period from 1 h after delivery of the placenta until 6 weeks postpartum. This period marks the establishment of a new phase of family life for women and their partners and the beginning of lifelong health for the newborns (1). Most neonatal deaths occur during this critical period in the lives of mothers and newborns (2).

In 2019, 2.4 million neonates died in their first month of life worldwide, with approximately 7,000 newborns dying every day. Despite a decrease in neonatal deaths from 5 million in 1990 to 2.4 million in 2019, the burden in Sub-Saharan Africa and Southern Asia remains high (3). Ethiopia, with 30 deaths per 1,000 live births, is one of the Sub-Saharan African countries with a high burden of neonatal mortality. Prematurity (21.8%), birth asphyxia (31.6%), and sepsis (18.5%) are the leading causes of neonatal deaths in Ethiopia (4).

The World Health Organization (WHO) proposed that by 2035, all countries will have achieved the goal of 10 or fewer newborn deaths per 1,000 live births and that they will continue to reduce death and disability, ensuring that no newborn is left behind. Improved skilled birth attendants, antenatal care visits, and neonatal care coverage, including home-based newborn care, could help achieve this goal. In addition, another WHO strategic objective to achieve reduced neonatal mortality is to leverage the power of parents, families, and communities (5).

Furthermore, all babies, regardless of birthplace, should receive neonatal care within the first 24 h of life, should be discharged from the birthing facility no sooner than 24 h after birth, and should receive at least four checkups within the first 6 weeks of life. Neonatal checkups are one of the milestones in the continuum of care required to achieve optimal maternal and child health. Neonatal care should be provided from a woman-friendly perspective. WHO emphasizes the importance of early post-hospital discharge follow-up by an experienced clinician for both women and their families (6) with the involvement of health workforce support, a woman-friendly perspective, and adequate infrastructure (7). As a result, psychosocial postpartum support programs have been promoted to improve maternal knowledge, attitudes, and skills related to parenting, maternal mental health, maternal quality of life, maternal physical health, timely recognition of danger signs, financial availability to arrange for transportation, affordability of health care costs, and accessibility to a health facility (8, 9).

Evidence shows that poor healthcare-seeking behaviors for neonatal illness, poorly perceived seriousness of the illness, cultural malpractices, and poor socioeconomic status are some of the barriers to utilization of neonatal care visits (10). Furthermore, delays in seeking healthcare, a lack of women’s autonomy to seek care, a lack of money, a heavy workload at home, deeply rooted cultural beliefs and rituals that guide care-seeking behavior, and restrictions on new mothers’ and newborns’ mobility for care-seeking all contribute to the problem (11, 12). Furthermore, the low coverage and poor quality of neonatal care provided reflect a persistently neglected component of maternity services and a gap in the continuum of neonatal care visit utilization (13). As a result, timely neonatal care visits and community-based intervention strategies, such as home visiting, health education, and counseling, are recommended (14, 15).

Maternal and child health care coverage is low in Ethiopia. Among pregnant women, an estimated 62% receive ANC and the rate of delivery in a health facility is 27%. The proportion of women receiving a postnatal check-up within 2 days of delivery is higher in urban areas (48%) than in rural areas (29%), lowest in Somalia (10%), and highest (74%) in Addis Ababa (16). Studies conducted in Ethiopia to identify the magnitude and determinants of postnatal care visits for neonates have inconsistent and inconclusive findings (17–22). ANC visit, place of delivery, residence, distance from the healthcare facility, educational status, age of the respondents, and mode of delivery were some of the factors that affect the postnatal checkup (17, 22–24). Therefore, this study helps estimate the overall rate of neonatal checkups and the factors associated with completing neonatal checkups in Ethiopia.

## Materials and Methods

### Data Source and Sampling Design

The data was taken from the 2016 EDHS report, which was conducted from January 18 to June 27, 2016. In Ethiopia, there are nine regional states and two city administrations. Each region was stratified into urban and rural areas. Stratified two-stage cluster sampling was performed. Samples of enumeration areas (EAs) were selected independently in each stratum in two stages. A total of 645 EAs (202 in urban areas and 443 in rural areas) were selected with a probability proportional to EA size. The full details are available from reference 16 (16).

Lists of households were used as a sampling frame for the second stage of household selection, and a fixed number of 28 households per cluster were chosen with an equal probability of systematic selection from the newly created household listing. The study population consisted of women with postpartum with a baby in the selected enumeration areas (EAs) and all postpartum mothers who had neonates in Ethiopia (16).

The data was extracted from 7,586 women with postpartum. The approval letter was obtained from the measure demographic and health survey (DHS), and the data set was downloaded from the DHS website^1^.

### Inclusion Criteria

All women with postpartum aged 15–49, who were either permanent residents or visitors who stayed in the selected households the night before the survey, were eligible.

Dependent variable: The primary outcome of interest was postnatal checkups for neonates (PNC). This variable was dummy-coded, so respondents who reported having PNC checkups for neonates were coded as “Yes,” while those who did not have PNC checkups were coded as “No.”

A postnatal care visit for a neonate was defined as at least one PNC visit within the first 42 days of the neonate’s postpartum period (25).

### Exposure Variables

Socio-demographic variables: Age of the mother’s residence (urban or rural), religion, marital status, and educational status (no education, primary education, secondary education, and above education).

Antenatal care visit (ANC) is defined based on self-reported frequency of any ANC services provided by skilled healthcare providers in the healthcare institutions, and categorized as “Yes” for any ANC visit and “No” for no ANC visit.

Place of delivery: Refers to whether the delivery was at a healthcare institution or home.

Fertility-related factors include the most recent child’s birth order (1st, 2nd, 3rd, 4th, etc.).

Mode of delivery: How did you give birth (vaginally, C/S, or instrumentally)?

Facility-related variables include the mother’s perceived distance from home to a health facility categorized as a “big problem” or “not a big problem.”

### Statistical Analysis

Data cleaning, recording, and analysis were carried out using SPSS statistical software version 24. Sample weight was applied to all analysis procedures to account for complex survey design and unequal probabilities of selection. A Rao-Scott chi-square test that adjusts for complex sample design was used to examine the bivariate associations between each covariate and the outcome variable. The data was a national survey data set with a hierarchical and cluster nature, which emphasizes the need for us to use a multilevel model of analysis. To use this model, the interclass calculation should be calculated, and be greater than 10%. The ICC in the current study was found to be 8.9%, which is lower than expected. As such, we used the binary logistic regression model. All variables with a *p*-value of ≤0.25 in the bivariable analysis were entered into the final model for multivariable analysis, and variables with *p*-values of <0.05 in the multivariable binary logistic regression model analysis were considered statistically significant. Finally, the result was presented using frequencies, tables, and texts.

## Results

### Socio-Demographic and Economic Characteristics of Mothers

The current EDHS analysis included 7,586 women who had a live birth in the 2 years preceding the survey. Almost half of the women (50.4%) were between the ages of 25 and 34, and 93.7% were married. The vast majority, 87.2%, came from rural areas. Seventy-eight percent of women were orthodox, while 37.2% were Muslim. Almost two-thirds of women, or 63.1%, were illiterate (Table 1).

**TABLE 1:** Socio-demographic characteristics of women with postpartum in Ethiopia (*N* = 7,586).

Variables	Frequency	Percentage (%)
**Age**		
15–24 years	1,804	23.8
25–34 years	3,823	50.4
>35 years	1,959	25.8
**Residence**		
Urban	969	12.8
Rural	6,617	87.2
**Marital status**		
Unmarried	481	6.3
Married	7,105	93.7
**Religion**		
Orthodox	2,881	38.0
Muslim	2,821	37.2
Protestant	1,651	21.8
Others	233	3.0
**Educational status**		
No education	4,788	63.1
Primary education	2,149	28.3
Secondary and above	649	8.6
**Wealth index**		
Poorest	1,649	21.7
Poorer	1,654	21.8
Middle	1,588	20.9
Richer	1,427	18.9
Richest	1,268	16.7

### Characteristics of Mothers and Neonatal Visit

More than half of women (58.1%) perceived the distance from nearby health facilities as a major problem for utilization of PNC checkups. Regarding ANC visits, 62.8% of women had antenatal care visits, and more than two-thirds (67.9%) of women gave birth at home. The majority (97.6%) of women gave birth vaginally and 95.7% had no awareness of danger signs (Table 2).

**TABLE 2:** Characteristics of the study sample and postnatal checkup for neonates (PNC) in Ethiopia (*N* = 7,586).

Variables	Total	PNC checkup		*P*-value*
	Frequency (%)	Yes	No	
Residence				<0.001
Urban	969 (12.8)	177 (18.3)	792 (81.7)	
Rural	6,617 (82.2)	455 (6.9)	6,162 (93.1)	
Educational status				<0.001
No education	4,788 (63.1)	315 (6.6)	4,473 (93.4)	
Primary education	2,149 (28.3)	198 (9.2)	1,951 (90.8)	
secondary and above	649 (8.6)	119 (18.4)	530 (81.6)	
Distance to health facility				<0.001
Big problem	4,404 (58.0)	249 (5.7)	4,154 (94.3)	
Not big problem	3,182 (42.0)	383 (12.0)	2,800 (88.0)	
ANC visit				<0.001
Yes	4,753 (62.8)	553 (11.6)	4,200 (88.4)	
No	2,818 (37.2)	79 (2.8)	2,739 (97.2)	
Mode of delivery				<0.001
Cesarean section	183 (2.4)	587 (7.9)	6,816 (92.1)	
Vaginal	7,403 (97.6)	45 (24.8)	138 (75.2)	
Awareness on neonatal danger sign				<0.001
Yes	330 (4.3)	107 (32.4)	223 (67.6)	
No	7,256 (95.7)	526 (7.2)	6,731 (92.8)	
Place of birth				<0.001
Health institution	2,401 (32.1)	363 (15.1)	2,038 (84.9)	
Home	5,071 (67.9)	252 (5.0)	4,819 (95.0)	
Birth order				0.002
1st	1,435 (18.9)	163 (11.4)	1,272 (88.6)	
2–4	3,188 (42.0)	265 (8.3)	2,923 (91.7)	
≥5	2,963 (39.1)	204 (6.9)	2,759 (93.1)	

*Rao-Scott chi-square p-value.

### The Magnitude of Postnatal Checkup

Six hundred thirty-two women, or 8.3%, had PNC for neonates within 42 days of giving birth.

### Determinants of Postnatal Checkup for Neonate

Binary logistic regression, in both bivariable and multivariable forms, was attempted. In a bivariable binary logistic regression analysis, residence, educational status, antenatal care visit, place of delivery, mode of delivery, awareness of neonatal danger signs, perceived distance from the health facility, and birth order were all the significant factors associated with postnatal checkups. The place of birth, ANC visit, and awareness of neonatal danger signs were all statistically significant predictors of postnatal checkups in the multivariable binary logistic regression analysis (Table 3).

**TABLE 3:** Determinants of PNC visit for neonates among women with postpartum in Ethiopia.

	Postnatal visit (Yes, No)	
	Crude odd ratio	Adjusted odd ratio
**Residence**		
Urban	3.03 (2.20, 4.18)	1.39 (0.91, 2.08)
Rural	1	1
**Educational status**		
No education	1	1
Primary education	1.43 (1.10,1.90)	0.87 (0.63,1.19)
Secondary and above	3.2 (2.30, 4.39)	1.0 (0.62,1.60)
**Distance to health facility**		
Big problem	1	1
Not big problem	2.28 (1.78, 2.91)	1.42 (1.06, 1.89)**
**ANC visit**		
Yes	4.60 (3.19, 6.57)	3.0 (1.99, 4.53)**
No	1	1
**Place of birth**		
Health institution	3.38 (2.60, 4.39)	1.55 (1.12, 2.15)*
Home	1	1
**Mode of delivery**		
Cesarean section	3.85 (2.29, 6.41)	0.98 (0.48, 2.03)
Vaginal	1	1
**Awareness of neonatal danger signs**		
Yes	6.13 (4.28, 8.77)	3.06 (2.09, 4.5)**
No	1	1
**Birth order**		
1st	1.73 (1.28, 2.35)	1.03 (0.69, 1.54)
2–4	1.23 (0.93,1.62)	0.88 (0.64,1.23)
≥5	1	1

*p-value < 0.009, **p-value < 0.001.

Women who perceived distance from healthcare institutions as not being a major issue were 1.42 times more likely than women who perceived distance as a major issue [AOR = 1.42; 95% CI: 1.06, 1.89]. Women who gave birth in a healthcare facility were 1.55 times more likely than women who gave birth at home to have postnatal care visits [AOR = 1.55; 95% CI: 1.12, 2.15]. Furthermore, women who had ANC visits were three times more likely to use neonatal care visits than women who did not have ANC visits [AOR = 3; 95% CI: 1.99, 4.53]. Women who were aware of neonatal danger signs were three times more likely to seek neonatal care than women who were unaware [AOR = 3.06; 95% CI: 2.09, 4.5] (Table 3).

## Discussion

Postnatal care visits are essential for increasing neonatal survival. This study aimed to determine the proportion of PNC and factors associated with PNC among women with postpartum in Ethiopia. We found that the postnatal checkup rate was 8.3%. The magnitude of PNC visits in the current study is lower than findings from Morocco (30.1%) (26), Nigeria (28.9%) (27), Ghana (62%) (28), India (29%) (29), and Nepal (43.2%) (30). One possible reason could be that the current study only included PNC, whereas other studies included postnatal checkups for mothers and neonates, which affects the overall magnitude of the postnatal care visit. Furthermore, it could be due to differences in the study setting, data collection method, and target population.

Women who perceived their distance from healthcare institutions as not being a major issue were 1.42 times more likely to use the neonatal care checkup than women who perceived distance from healthcare institutions as being a major issue. The finding is similar to studies conducted in Indonesia (31) and developing countries (32). This could be because women who live a long distance away from a healthcare facility may have difficulty obtaining transportation (33, 34). Furthermore, it causes a maternal delay in seeking healthcare services (35).

Women who had ANC visits were three times more likely than women who did not have ANC visits to use a PNC checkup. The finding is supported by studies conducted in Ethiopia (22, 27, 28, 30, 36, 37). Previous studies suggest that antenatal care visits increase mothers’ birth preparedness and complication readiness (38). Moreover, women who had antenatal care visits were more knowledgeable about maternal and neonatal complications (39). This increased knowledge could explain increased postnatal care checkups.

The study revealed that women who gave birth at healthcare institutions were 1.55 times more likely to have a PNC visit than women who gave birth at home. This is in line with other studies conducted in Ethiopia (19, 37, 40, 41), and Nepal (30). Giving birth in a health facility increases both women’s awareness and knowledge of the benefits of neonatal checkups, provides better information on postpartum complications, increases access to healthcare services, and increases the mother’s health-seeking behavior. Evidence suggests that education about postnatal care schedules leads to an increase in PNC visits (42).

Women who were aware of neonatal danger signs were 3.06 times more likely than women who were not aware of neonatal danger signs to have a PNC checkup for neonates. The findings are consistent with other studies conducted in various parts of the world (43, 44). This could be related to enhanced awareness of mothers on neonatal danger signs, which may increase those who seek healthcare (45). Understanding neonatal danger signs also assists women in identifying early warning signs of a neonatal problem, which increases neonatal care-seeking behaviors.

A strength of this study was the data taken from a large and representative sample size. A limitation of this study is that, because of its cross-sectional nature, it does not demonstrate a cause-and-effect relationship between the variables examined and the completion of neonatal checkups. Survey responses may also have been affected by social desirability bias.

## Conclusion

In this study, the national PNC completion rate was very low at just 8.3%. PNC visit was associated with perceived distance from healthcare institutions, institutional delivery, having ANC visits, and being aware of neonatal danger signs. Our findings suggest that improving ANC visits, delivery in healthcare facilities, maternal awareness of neonatal danger signs, and access to health care, including through home visits, may improve rates of PNC in Ethiopia. Moreover, emphasis should be given to community-based newborn care packages, which have been implemented in Ethiopia.

## Data Availability Statement

The raw data supporting the conclusions of this article will be made available by the authors, without undue reservation.

## Ethics Statement

Permission was obtained to use the EDHS data from the measure DHS International Program, and approval data was also obtained. All methods were performed in accordance with the relevant guidelines and regulations. Informed consent was given.

## Author Contributions

BB, DB, DM, EC, and WA designed the study, interpreted the results, and prepared the manuscript. BB, DB, AE, DA, SD, GN, EA, SN, YT, and DJ analyzed, interpreted, and wrote the manuscript. TM reviewed and edited the manuscript. All authors were involved in design, data interpretation, and reviewed the manuscript.

## Conflict of Interest

The authors declare that the research was conducted in the absence of any commercial or financial relationships that could be construed as a potential conflict of interest.

## Publisher’s Note

All claims expressed in this article are solely those of the authors and do not necessarily represent those of their affiliated organizations, or those of the publisher, the editors and the reviewers. Any product that may be evaluated in this article, or claim that may be made by its manufacturer, is not guaranteed or endorsed by the publisher.

## Footnotes

1
http://www.dhs.com

## Abbreviations

AOR: adjusted odd ratio
ANC: antenatal care visit
CI: confidence interval
DHS: Demographic and Health Survey
EDHS: Ethiopian Demographic and Health Survey
PNC: postnatal care visit
SDG: sustainable development goal
WHO: World Health Organization.
